# Malignant Transformation in a Mature Teratoma with Metastatic Deposits in the Omentum: A Case Report

**DOI:** 10.1155/2012/568062

**Published:** 2012-10-03

**Authors:** Shramana Mandal, Bhawana A. Badhe

**Affiliations:** Department of Pathology, JIPMER, Puducherry 605006, India

## Abstract

Malignant transformation of a mature cystic teratoma (MCT) is a very rare complication with an incidence of 0.17–2%;. The most common form of malignant transformation of the MCT is squamous cell carcinoma. Other tumors arising in MCT include basal cell carcinoma, sebaceous tumor, malignant melanoma, adenocarcinoma, sarcoma, and neuroectodermal tumor. However malignant transformation with metastatic deposits in the omentum is extremely rare. The present case highlights the rarity of the occurrence of an omental deposits in a case of mature cystic teratoma with malignant transformation.

## 1. Introduction

Malignant transformation in a mature cystic teratoma (MCT) is extremely rare [1]. Its incidence is 0.17–2% of all cases. MCT with deposits in the omentum is even rarer. The exact incidence is not known. The prognosis of malignant transformation of the MCT is very poor [2]. Extensive literature search has revealed only 3 such cases. We report the fourth case of MCT with omental deposits of squamous cell carcinoma in a 56-year-old female.

## 2. Case Report

**Figure 1:**
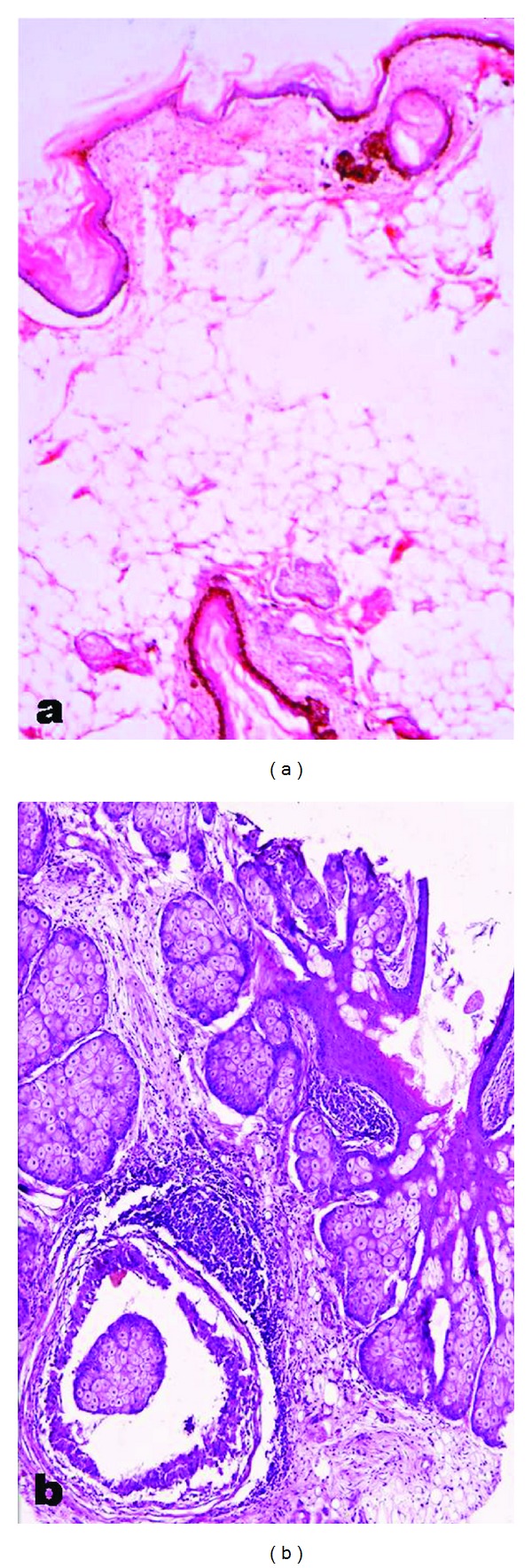
Shows typical features of a mature cystic teratoma. Cyst wall being composed of ectodermal elements.

**Figure 2:**
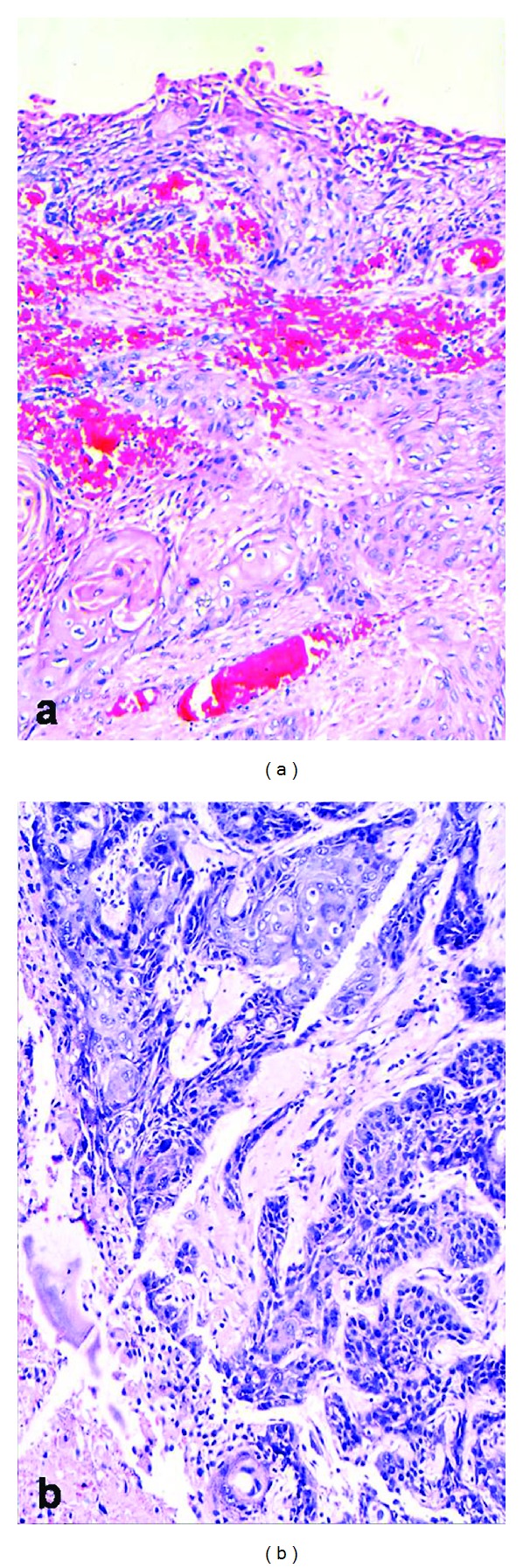
Section from the solid areas showed well-differentiated squamous cell carcinoma arising from the surface epithelium.

A 56-year-old female presented with history of abdominal distension and pain for 5 months. On per vaginal examination, a mass was felt arising from the pelvis, which was cystic, nontender, and mobile. The border of the mass was not well defined. Grossly a multiloculated ovarian mass was received measuring 13 × 10 × 9 cm in size with an area of capsular breech. Cut surface was multiloculated, cystic, filled with grumous material, hair, and tooth. A single solid island measuring 4 × 3 × 2 cm was also identified, the cut surface of which was grey white and firm. Microscopical sections showed typical features of a mature cystic teratoma. The cyst wall was composed of ectodermal elements (Figure 1). Section from the solid areas showed well-differentiated squamous cell carcinoma arising from the surface epithelium (Figure 2). Omentum showed deposits of well-differentiated squamous cell carcinoma. A final diagnosis of malignant transformation of well-differentiated squamous cell carcinoma in a mature cystic teratoma with omental deposits was made.

## 3. Discussion 

The mature cystic teratoma (MCT) is the most common germ cell tumor of ovary, comprising of more than 10–20% of all ovarian neoplasms [1]. It is composed of a well-differentiated derivation of all the three germ cell layers, that is, endoderm, mesoderm, and ectoderm. Mature cystic teratomas can occur at any age; however, they are very common in women of childbearing age and occur bilaterally in 10–17% of patients [2]. The complications associated with cystic teratoma cases include torsion (16%), malignant degeneration (2%), rupture into adjacent organs (1-2%), and infection (1%) [3].

Malignant change is rarely recognized preoperatively. Most patients with such tumours have symptoms of abdominal pain and mass, which do not differ from those of uncomplicated mature cystic teratomas such as abdominal pain and mass. These tumours range in sizes from 30 mm to 400 mm [1, 4]. The risk of malignancy is related to age and is substantially greater in postmenopausal women, the highest incidence being in the fifth and sixth decades of life [5].

Malignant transformation of an MCT is an uncommon complication occurring in approximately 0.17–2% of all mature cystic teratomas [4, 6]. Although any of the constituent tissues of teratoma has the potential to undergo malignant transformation, squamous cell carcinoma is the most commonly associated cancer. However malignant transformation with metastasis to the omentum is extremely rare. 

Rarely, other tumors (0.2–1.4%) can arise in an MCT like adenocarcinoma, basal cell carcinoma, adenosquamous carcinoma, thyroid carcinoma, sebaceous carcinoma, malignant melanoma, sarcoma, carcinoid tumor, and neuroectodermal tumour [6]. 

The low incidence of secondary malignant transformation of mature cystic teratoma explains why a few reports have been published. It has been thought that squamous cell carcinoma in mature cystic teratoma arises from metaplastic squamous epithelium [7]. Furthermore, high-risk human papillomavirus infection has also been thought to be associated with ovarian squamous-cell carcinoma [8]. Mostly mature cystic teratomas are detected 15–20 years before they undergo secondary malignant transformation. Cytogenetic abnormalities might precede histological changes, and prolonged exposure to various carcinogens in the pelvic cavity might cause the malignant changes in mature tissue. Thus, squamous-cell carcinoma in mature cystic teratoma is more common in postmenopausal patients. Pelvic ultrasonography can help in early detection of these tumours in women of childbearing age [9]. 

Grossly these tumours show presence of nodular, papillary, or cauliflower-like growths protruding into the cyst cavities or nodules or plaques within the cyst walls along with areas of capsular invasion.

Serum tumour markers like squamous cell carcinoma antigen, CA125, CA19-9, and CEA are useful in distinguishing mature cystic teratoma from malignant transformation. Tissue polypeptide antigen and macrophage colony stimulating factor may also help to predict malignant transformation in this tumour [4].

Because all preoperative diagnostic procedures can sometimes be unreliable in excluding malignant disease, all mature cystic teratomas in women more than 30 years that show unusual adherence, solid or firm, friable, myxomatous, or variegated areas should arouse suspicion. Hence, surgical approach should be chosen carefully based on the results of clinical and imaging investigations as well as tumour marker profiles [4]. 

Removal of the entire tumour, in accordance with oncosurgical treatment principles, is essential, following which complete cytoreduction can further improve the outcome in these patients. Alkylating drugs can be given for chemotherapy regimens, whereas, radiotherapy can lead to greater morbidity [4, 10].

 To conclude, early detection and complete surgical resection are important for long-term survival. Adequate sampling is essential in these ovarian tumors to establish their teratomatous origin and avoid an erroneous diagnosis.
